# Supplementary material for: Brain disconnections link structural connectivity with function and behaviour
Source: Nat Commun. 2020 Oct 9;11:5094. doi: 10.1038/s41467-020-18920-9 (PMC7547734; doi:10.1038/s41467-020-18920-9)
Supplement: Supplementary file 3 — Reporting Summary [file 41467_2020_18920_MOESM3_ESM.pdf]

nature research

Corresponding author(s): Michel Thiebaut de Schotten

Last updated by author(s): 17/9/20

Reporting Summary

Nature Research wishes to improve the reproducibility of the work that we publish. This form provides structure for consistency and transparency in reporting. For further information on Nature Research policies, seeAuthors & Referees and theEditorial Policy Checklist .

Statistics

For all statistical analyses, confirm that the following items are present in the figure legend, table legend, main text, or Methods section.

n/a	Confirmed	
☐	☒	The exact sample size (n) for each experimental group/condition, given as a discrete number and unit of measurement
☐	☒	A statement on whether measurements were taken from distinct samples or whether the same sample was measured repeatedly
☐	☒	The statistical test(s) used AND whether they are one- or two-sided<br>*Only common tests should be described solely by name; describe more complex techniques in the Methods section.*
☐	☒	A description of all covariates tested
☐	☒	A description of any assumptions or corrections, such as tests of normality and adjustment for multiple comparisons
☐	☒	A full description of the statistical parameters including central tendency (e.g. means) or other basic estimates (e.g. regression coefficient) AND variation (e.g. standard deviation) or associated estimates of uncertainty (e.g. confidence intervals)
☐	☒	For null hypothesis testing, the test statistic (e.g. F, t, r) with confidence intervals, effect sizes, degrees of freedom and P value noted<br>*Give P values as exact values whenever suitable.*
☒	☐	For Bayesian analysis, information on the choice of priors and Markov chain Monte Carlo settings
☒	☐	For hierarchical and complex designs, identification of the appropriate level for tests and full reporting of outcomes
☐	☒	Estimates of effect sizes (e.g. Cohen's d, Pearson's r), indicating how they were calculated

Our web collection on statistics for biologists contains articles on many of the points above.

Software and code

Policy information about availability of computer code

Data collection	no software was used.
Data analysis	Open software BCBtoolkit 4.1, open software FSL 6.0, Python 3, NiBabel 3.1.1, scikit-learn 0.23, Tract Querier, Surf Ice 2 September 2019.<br>Code available with the manuscript and on demand to the authors (michel.thiebaut@gmail.com).<br>https://github.com/chrisfoulon/BCBlib/blob/devel/bcblib/scripts/generate_synth_lesions.py<br>https://github.com/chrisfoulon/BCBlib/blob/devel/bcblib/scripts/pick_up_matched_synth_lesions.py

For manuscripts utilizing custom algorithms or software that are central to the research but not yet described in published literature, software must be made available to editors/reviewers. We strongly encourage code deposition in a community repository (e.g. GitHub). See the Nature Research guidelines for submitting code & software for further information.

Data

Policy information about availability of data

All manuscripts must include a data availability statement. This statement should provide the following information, where applicable:

- Accession codes, unique identifiers, or web links for publicly available datasets
- A list of figures that have associated raw data
- A description of any restrictions on data availability

Source data are provided with this paper. The two sets of component maps and the atlas of white matter function (original and replication) are available at https://identifiers.org/neurovault.collection:7735
The atlas of white matter function is also available at
A to C terms: https://identifiers.org/neurovault.collection:7756
D to H terms: https://identifiers.org/neurovault.collection:7757

I to N terms: https://identifiers.org/neurovault.collection:7758
O to R terms: https://identifiers.org/neurovault.collection:7759
S to U terms: https://identifiers.org/neurovault.collection:7760
V to Z terms: https://identifiers.org/neurovault.collection:7761
The raw dataset analysed in the current study are available at https://www.humanconnectome.org (7T diffusion data), http://www.neurosynth.org (metanalytic functional MRI maps). In addition, processed data are available at http://www.bcblab.com/BCB/Opendata.html and https://osf.io/5zqwg/ and on request to the corresponding author michel.thiebaut@gmail.com.

Field-specific reporting

Please select the one below that is the best fit for your research. If you are not sure, read the appropriate sections before making your selection.

- [x] Life sciences
- [ ] Behavioural & social sciences
- [ ] Ecological, evolutionary & environmental sciences

For a reference copy of the document with all sections, see nature.com/documents/nr-reporting-summary-flat.pdf

Life sciences study design

All studies must disclose on these points even when the disclosure is negative.

Sample size	n = 2666 lesions (1333 stroke and 1333 control synthetic lesions). No sample size calculation was performed. We chose the biggest stroke dataset ever reported to our knowledge in order to model stroke. This sample size was powered enough to allow us to model >90% of the variance of stroke disconnection.
Data exclusions	For the split half approach 1 subject was randomly excluded to ensure that the two datasets include the same number of participants (n = 666)
Replication	We chose a split half approach to test the reproducibility of the component maps and the atlas of white matter function. Split half approach is the strongest replication approach when another independent dataset is not available. Reproducibility was assessed using Pearson correlation. All attempts at replication were successful
Randomization	Synthetic lesions were paired in size and brain hemisphere. The allocation to the group for the replication was random.
Blinding	the study is data driven, data are fully available for replication, therefore blinding was not relevant here.

Reporting for specific materials, systems and methods

We require information from authors about some types of materials, experimental systems and methods used in many studies. Here, indicate whether each material, system or method listed is relevant to your study. If you are not sure if a list item applies to your research, read the appropriate section before selecting a response.

Materials & experimental systems

n/a	Involved in the study
x	Antibodies
x	Eukaryotic cell lines
x	Palaeontology
x	Animals and other organisms
x	Human research participants
x	Clinical data

Methods

n/a	Involved in the study
x	ChIP-seq
x	Flow cytometry
	x MRI-based neuroimaging

Magnetic resonance imaging

Experimental design

Design type	Event related and block design meta-analysis
Design specifications	task-related fMRI activation meta-analysis (neurosynth.org)
Behavioral performance measures	590 cognitive functions

Acquisition

Imaging type(s)	Diffusion
Field strength	7T (diffusion)
Sequence & imaging parameters	Each diffusion-weighted imaging consisted of a total of 132 near-axial slices acquired with an acceleration factor of 3, isotropic (1.05 mm3) resolution and coverage of the whole head with a TE of 71.2 ms and with a TR of 7000 ms.
Area of acquisition	Brain

Diffusion MRI ☒ Used ☐ Not used

Parameters: At each slice location, diffusion-weighted images were acquired with 65 uniformly distributed gradients in multiple Q-space shells and 6 images with no diffusion gradient applied.This acquisition was repeated four times with a b-value of 1000 and 2000 s mm−2 in pairs with anterior-to-posterior and posterior-to-anterior phase-encoding directions.

Preprocessing

Preprocessing software	BCBtoolkit (disconnectome maps and normalisation of the tractography)<br>FSL (randomize)
Normalization	linear and non linear diffeomorphic normalisation using BCBtoolkit (that actually uses the code of Advanced Normalisation Tools) applied to the fibre density map to register it with the MNI152 fibre density map template (homade, available as part of BCBtoolkit), the same deformation is applied to the streamlines of the tractography using Tract Querier
Normalization template	MNI152 fibre density map template
Noise and artifact removal	In short, the susceptibility-induced off-resonance field was estimated from pairs of images with diffusion gradient applied with distortions going in opposite directions and corrected for the whole diffusion-weighted dataset using TOPUP. Subsequently, motion and geometrical distortion were corrected using the EDDY tool as implemented in FSL.
Volume censoring	no censoring

Statistical modeling & inference

Model type and settings	Multivariate (for the component maps) and univariates (for the Atlas of White Matter Function) regressions.
Effect(s) tested	Component maps prediction: We assessed the independent contributions of the components to disconnections in the white matter (component maps - multivariate).<br>Functional activation prediction: We assessed the contribution of the component maps voxels to each meta analytic maps (Atlas of White Matter Function - univariate)

Specify type of analysis: ☐ Whole brain ☐ ROI-based ☒ Both

Anatomical location(s): We used the MultiModal Parcellation of Matt Glasser (Nature 2016) to characterise profiles of disconnection.

Statistic type for inference (See Eklund et al. 2016)	Voxel-wise
Correction	FWE

Models & analysis

n/a	Involved in the study
☒	☐ Functional and/or effective connectivity
☒	☐ Graph analysis
☐	☒ Multivariate modeling or predictive analysis

Multivariate modeling and predictive analysis:

Data compression: PCA using a covariance matrix and varimax rotation
(with a maximum of 500 iterations for convergence) was applied to matrices of disconnection in order toestimate the number of principal components.
Component maps prediction: We used a permuted (n = 1000) multiple regression to statistically assess the relationship between each component and voxel in the white matter. To do so, we used the component score as an independent variable and the voxel probability of disconnection in the stroke disconnectome maps as dependant variables.
Functional activation prediction: A permuted (n = 1000) linear regression was computed between the correlation value of each task-related fMRI meta-analytic map as an independent variable and each component maps voxels as a dependant variable.
