# Supplementary material for: Brain disconnections link structural connectivity with function and behaviour
Source: Nat Commun. 2020 Oct 9;11:5094. doi: 10.1038/s41467-020-18920-9 (PMC7547734; doi:10.1038/s41467-020-18920-9)
Supplement: Supplementary file 4 — Description of Additional Supplementary Files [file 41467_2020_18920_MOESM4_ESM.pdf]

Description of Additional Supplementary Files

File Name: Supplementary Data 1
Description: Full list of the 46 components pattern of disconnection (components scores)

File Name: Supplementary Data 2
Description: Complete report of all task-related functional imaging correlations for each component
